# Upadacitinib as rescue therapy for corticosteroid failure acute severe ulcerative colitis: an Asian experience from Taiwan

**DOI:** 10.1007/s00384-025-04825-w

**Published:** 2025-02-11

**Authors:** Chen-Shuan Chung, Wei-Wei Lee, Puo-Hsien Le

**Affiliations:** 1 Division of Gastroenterology and Hepatology, Department of Internal Medicine, Far Eastern Memorial Hospital, New Taipei City, Taiwan; 2 College of Medicine, Fu Jen Catholic University, New Taipei City, Taiwan; 3 Taiwan Association for the Study of Intestinal Diseases (TASID), Taoyuan, Taiwan; 4 Department of Gastroenterology and Hepatology, Linkou Chang Gung Memorial Hospital, Taoyuan, Taiwan; 5 Inflammatory Bowel Disease Center, Linkou Chang Gung Memorial Hospital, 5, Fu-Hsin Street, Guei-Shan District, Taoyuan, 33305 Taiwan

**Keywords:** Acute severe ulcerative colitis, Upadacitinib, Small molecule drugs, Salvage therapy

## Abstract

**Purpose:**

Acute severe ulcerative colitis (ASUC) is a medical emergent condition, and approximately one-third of patients with ASUC do not respond to corticosteroid. Whether small molecule drugs are efficient and safe for salvage therapy of ASUC is not well-understood.

**Methods:**

Consecutive patients with ASUC who failed responding to first-line corticosteroid were retrospectively enrolled. Clinical, laboratory, endoscopic, and pathological data were analyzed.

**Results:**

Five Asian male patients (median age of 38.9 years old) with ASUC who received salvage therapy with upadacitinib were enrolled. The mean (± standard deviation) disease duration was 3.44 (± 3.30, 0.53 ~ 7.88) years. Baseline Montreal disease extent, C-reactive protein, and erythrocyte sedimentation rate were four (80%) E3 and one (20%) E2 disease, 40.54 (± 74.26) mg/dl, and 24.50 (± 19.09) mm/h, respectively. Daily upadacitinib 45 mg was prescribed for 56 days in all patients. Clinical response, clinical remission, corticosteroid-free remission, and endoscopic improvement at weeks 8 and 12 were achieved in five (100%) and five (100%), four (80%) and five (100%), four (80%) and five (100%), and four (80%) and five (100%) patients, respectively. One (20%) patient achieved histo-endoscopic remission at week 24. None of them had re-hospitalization or colectomy during the follow-up period but one patient developed penile simplex-1 infection which resolved after topical antiviral ointment without upadacitinib discontinuation.

**Conclusions:**

Upadacitinib is an efficient salvage therapy for patients with ASUC. Further investigations are essential to assess long-term durability, safety profile, and viability as a bridging regimen in the treatment of ASUC.

## Background

Ulcerative colitis (UC) is a chronic inflammatory condition of colon with several cumbersome complications when inflammation is not well controlled. The disease course varies between individuals, reflecting a diverse range of clinical outcomes [1]. Despite improvements in advanced therapies, disease flare requiring hospitalization occurs in 15 ~ 25% of UC patients with 20% surgical needs, 30 ~ 40% comorbidities, and 1% mortalities [1, 2]. Once confirmatory diagnosis of acute severe UC (ASUC) is made, hospital admission and intravenous corticosteroids should not be delayed [1, 2]. Notwithstanding, 30 ~ 40% of them might not respond to systemic corticosteroids with significant morbidities and mortalities prompting rescue medical therapies, including infliximab and calcineurin inhibitors, or surgical colectomy [1, 2]. Among advance therapies for UC, Janus kinase inhibitors (JAKIs) have been proven as highly effective medications for both induction and maintenance therapy of moderate-to-severe UC [3]. These small molecule drugs are one of the promising options for ASUC management notably because of its rapid onset of action. Tofacitinib, a pan-JAKI, has been reported for treating ASUC refractory to first-line steroid or rescue infliximab therapy [4]. However, infectious adverse events (AEs) are noticed in these studies [4]. Upadacitinib, which is engineered to block JAK-1 selectively without inhibiting JAK-2, JAK-3, and tyrosine kinase 2 (TYK2), has a more favorable safety profile. Its rapidity in inducing clinical remission potentiates its crucial role in rescue therapy of ASUC. Herein, we report the first investigation in Asian population to use upadacitinib as salvage therapy in steroid refractory ASUC patients.

## Methods

It was a multicenter retrospective study conducted at two tertiary centers (Far Eastern Memorial Hospital and Linkou Chang Gung Memorial Hospital) in Taiwan. Patients with ASUC who failed to respond to intravenous corticosteroid and other salvage therapy were consecutively enrolled for study. Clinical, biochemical, and endoscopic data of those who received upadacitinib treatment were analyzed. It was approved by the Research Ethics Review Committees of the study institutes (FEMH-107145-F, Chang Gung Medical Foundation 202400030B0), and all study protocols were performed in accordance with the Committee on Publication Ethics guideline and the Declaration of Helsinki for medical research. The written informed consents were provided to all patients who were eligible for enrollment.

Clinical response and endoscopic improvement were defined as ≥ 3 points in Ulcerative Colitis Disease Activity Index (UCDAI) and ≥ 1 reduction in Mayo endoscopic subscore (MES) from baseline, respectively. Clinical remission was considered as UCDAI score ≤ 1 after 8 weeks with subscores of 0 for rectal bleeding and stool frequency. Histo-endoscopic remission was defined as endoscopic Mayo subscore ≤ 1 plus Nancy histological index ≤ 1.

Continuous and categorical variables were expressed as mean (± standard deviation) and count (%), respectively. The statistical analysis was performed using STATA software (version 14.0; Stata Corp, College Station, TX, USA).

## Results

**Fig. 1:**
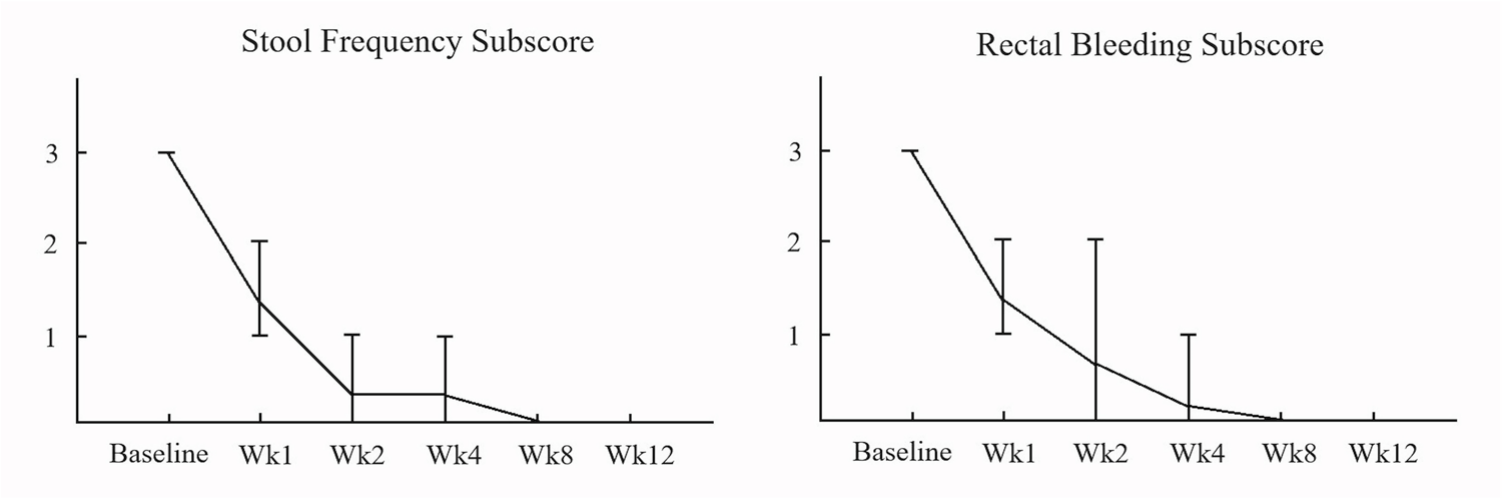
Line chart of stool frequency and rectal bleeding subscore after upadacitinib salvage therapy in terms of patient-reported outcome

**Fig. 2:**
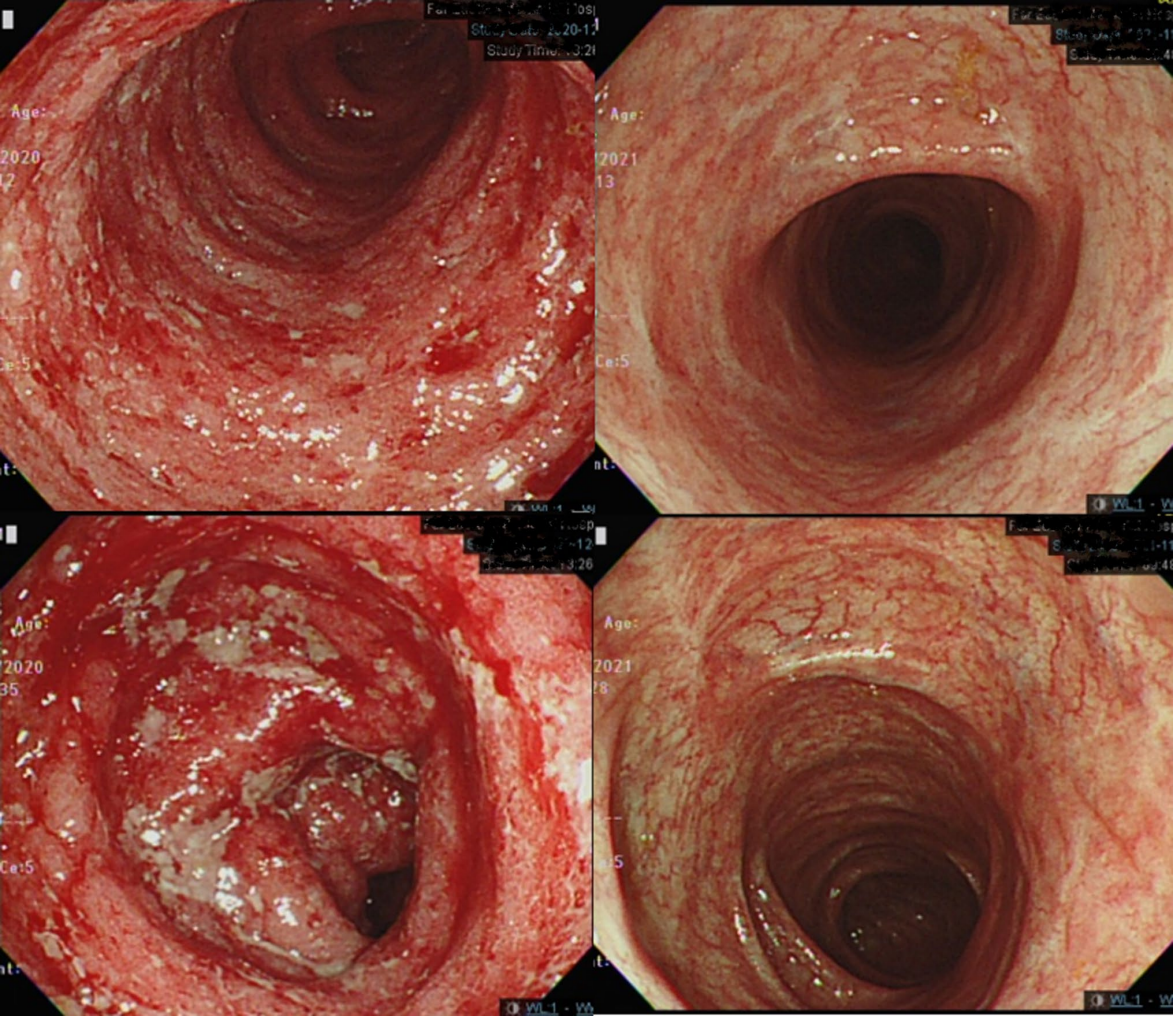
*Left panel*: Mayo endoscopic subscore 3 before medical treatment. *Right panel*: Mayo endoscopic subscore 0 after 12 weeks treatment with upadacitinib salvage therapy

**Fig. 3:**
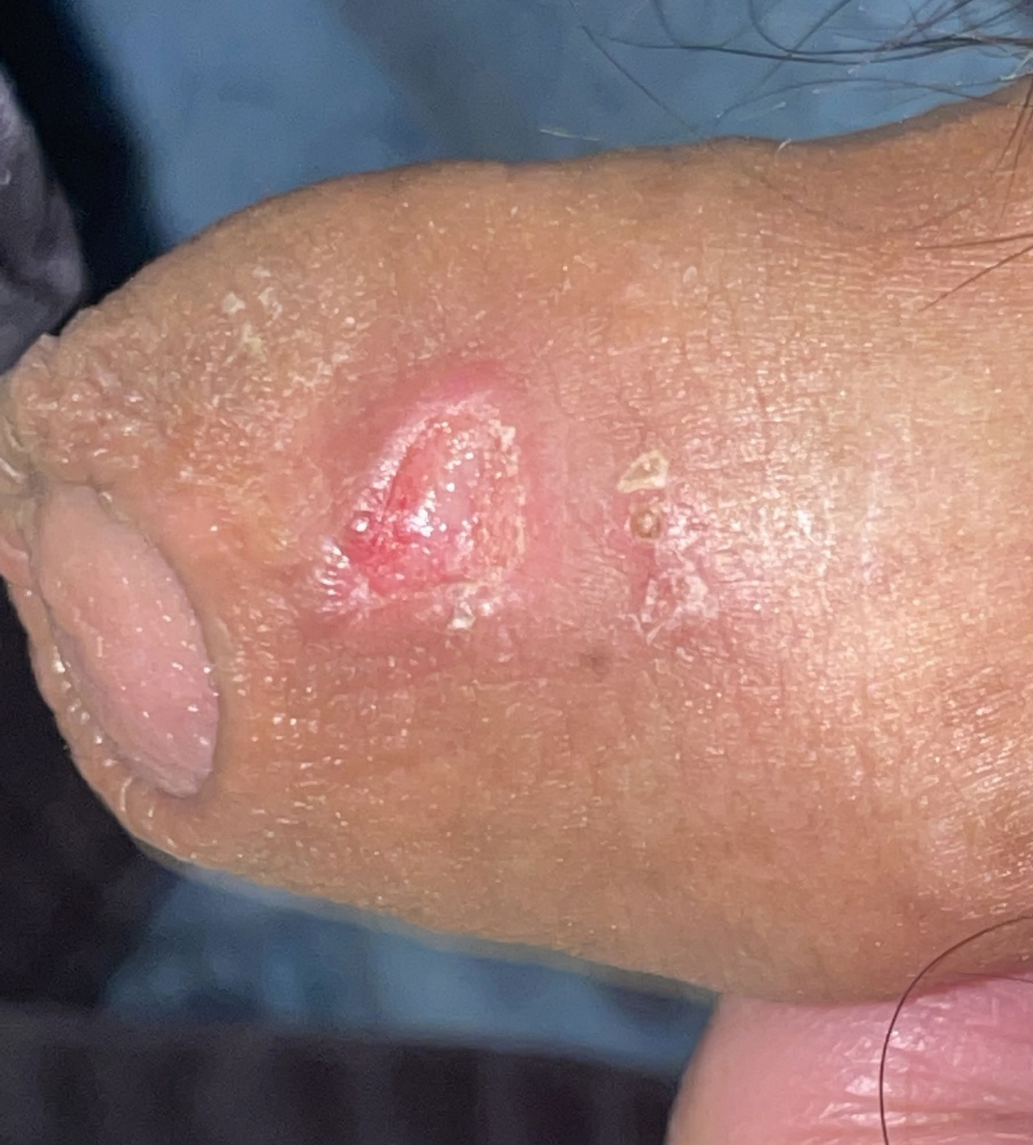
Penile herpes simplex virus-1 infection at week 2 of upadacitinib salvage therapy

Five male patients who were admitted for ASUC were enrolled (Table 1). The median age was 38.9 years old while mean (± standard deviation, range) disease duration was 3.44 (± 3.30, 0.53 ~ 7.88) years. Four (80%) patients had baseline Montreal classification E3 disease and one (20%) patient with E2 disease. Only one (20%) patient was biologics-naïve while one (20%) patient exposed to four, two (40%) to three, and one (20%) to two kinds of biologics previously. The C-reactive protein level, erythrocyte sedimentation rate, and albumin level were 40.54 (± 74.26, 2.30 ~ 172.73) mg/dl, 24.50 (± 19.09, 7 ~ 49) mm/h, and 3.42 (± 0.41, 3.10 ~ 4.10) g/dl, respectively. All (100%) patients had partial Mayo score of 9 and endoscopic subscore of 3. Among them, four (80%) patients failed to respond to 3-day intravenous corticosteroid and one patient (20%) did not respond to 3-day corticosteroid and infliximab rescue therapy at 7 days. Daily upadacitinib 45 mg was prescribed for salvage therapy in all patients. Patient-reported outcome including stool frequency and rectal bleeding improved at week 2 after upadacitinib therapy (Fig. 1). Clinical response, clinical remission, corticosteroid-free remission, and endoscopic improvement at week 8/12 were achieved in five (100%)/five (100%), four (80%)/five (100%), four (80%)/five (100%), and four (80%)/five (100%) patients, respectively (Fig. 2). One (20%) patient achieved histo-endoscopic remission with endoscopic Mayo subscore 0 and Nancy histological index score 1. There was no patient experiencing re-hospitalization or colectomy within 24 weeks of ASUC onset. Regarding upadacitinib-related adverse events, case 3 developed herpes simplex-1 (HSV-1) infection upon prepuce after 2 weeks (Fig. 3). Upadacitinib was not discontinued, and the vesicular lesions improved after topical therapy with acyclovir ointment.

**Table 1:** Baseline clinical, biochemical, and endoscopic data of the enrolled subjects with acute severe ulcerative colitis

Case	Disease duration (years)/disease extent (Montreal)	Biologics exposure history	ESR (mm/h)/CRP (mg/dl)/albumin (g/dl)	MES/UCEIS/UCDAI scores	Failure to medication
1	6.01/E3	Four biologics	12/2.30/3.20	3/6/12	Steroids
2	0.96/E3	Naïve	49/20.22/3.10	3/8/12	Steroids
3	7.88/E2	3 biologics	30/3.73/3.20	3/8/12	Steroids
4	0.53/E3	2 biologics	7/172.73/4.10	3/8/12	Steroids
5	1.84/E3	3 biologics	NA/3.72/3.49	3/8/12	Steroids + IFX

Abbreviations: *CRP* C-reactive protein, *ESR* erythrocyte sedimentation rate, *IFX* infliximab, *MES* Mayo endoscopic subscore, *UCDAI* Ulcerative Colitis Disease Activity Index, *UCEIS* Ulcerative Colitis Endoscopic Index of Severity

## Discussion

The overall corticosteroid response rate in ASUC patients was about 60 ~ 70% [1, 2]. For those who do not respond to a minimum of 3 days steroid, rescue medical therapy, including cyclosporine and infliximab which are similar in efficacy and safety according to the results of randomized controlled trials, or surgical colectomy should be considered without delay [1, 2, 5]. However, adverse events of cyclosporine, such as impaired renal and hepatic function, hypertension, and drug-drug interactions, and the immunogenicity and opportunistic infection while using infliximab should be cautiously aware of. The pharmacokinetic advantages of JAKI for the management of UC include the rapid absorption and onset of action and short half-life with fast clearance as well as the low immunogenicity [6]. They are less affected by hypoalbuminemia and protein leakage leading to drug loss in stool compared to infliximab [7]. It has proven that JAKIs are highly effective in induction of moderate-to-severe UC according to clinical trials and they are deemed as the best performing agent for the inducing clinical remission when compared to other biologics in a meta-analysis [3, 8]. A case series using high-dose (10 mg three times daily) tofacitinib for the management of 11 ASUC patients who were refractory to steroid and infliximab reported 90.9% clinical and biochemical response rate and 18.2% early colectomy rate [9]. Meta-analyses showed a pooled 90-day and 6-month colectomy-free rate of 79.9 ~ 86% and 69 ~ 71.6%, respectively, after rescue therapy using tofacitinib [4]. Infections are the most common (8.8 ~ 11.9%) AE from tofacitinib while herpes zoster and venous thromboembolism rate of 1.4% and 0.7%, respectively [4]. Upadacitinib is a selective JAKI with high affinity to JAK-1 which might provide better treatment efficacy and a favorable safety profile than pan-JAKI. A case series using upadacitinib 45 mg daily for salvage therapy after steroid in six ASUC patients showed clinical response rate, corticosteroid-free clinical remission rate, and colectomy rate of 100%, 66.7%, and 16.7%, respectively [10]. Another case series of four ASUC patients who were treated with upadacitinib 45 mg daily after failing steroid and infliximab rescue therapy disclosed clinical response rate and colectomy rate of 75% and 25%, respectively [8]. In our case report, a favorable short-term outcome was noticed after upadacitinib salvage therapy with clinical response, clinical remission, and corticosteroid-free remission of 100%, 80%, and 80%, respectively, at week 8, and all endpoints with 100% at week 24. In addition, one (20%) patient had histo-endoscopic remission. None of them had re-hospitalization or colectomy, but one patient experienced genital HSV-1 infection which resolved after topical antiviral medication without discontinuation of upadacitinib.

In conclusion, small molecule drugs, JAKI, are promising salvage therapy for patients with ASUC. Nevertheless, more investigations about long-term durability, safety, and appropriate bridging regimens after high-dose induction therapy are warranted.

## Data Availability

No datasets were generated or analysed during the current study.
